# Tandem Disc Herniation of the Lumbar and Cervical Spine: Case Series and Review of the Epidemiological, Pathophysiological and Genetic Literature

**DOI:** 10.7759/cureus.4081

**Published:** 2019-02-16

**Authors:** Alessandro Siccoli, Victor E Staartjes, Marlies P De Wispelaere, Pieter-Paul A Vergroesen, Marc L Schröder

**Affiliations:** 1 Neurosurgery, Bergman Clinics, Amsterdam, NLD; 2 Clinical Informatics, Bergman Clinics, Amsterdam, NLD; 3 Orthopedic Surgery, University Medical Center, Utrecht, NLD

**Keywords:** tandem, disc degeneration, intervertebral, genetics, multisegment, disc herniation, pathophysiology, biomechanics, degenerative disc disease, epidemiology

## Abstract

Introduction

Lumbar disc herniation (LDH) and cervical disc herniation (CDH) represent a relevant public health problem. Patients with symptomatic tandem herniations of the cervical and lumbar spine are rare and not described in the literature. In these patients, certain variables may predispose the development of disc herniation which could increase the understanding of the development of disc herniations. Our aim is to present the first case series of tandem disc herniation, and to elucidate whether tandem herniation is attributable to a certain propensity for disc herniation or not.

Methods

From a prospective registry, patients with symptomatic tandem disc herniations were included, and the literature was reviewed on the comparative pathophysiology, genetics, and epidemiology of disc herniation and disc degeneration.

Results

Out of 3,156 patients with disc herniations in our registry, 16 presented with symptomatic tandem LDH and CDH that required discectomy. Therefore, we estimate the incidence of tandem disc herniation at 0.51% (95% confidence interval (CI): 0.26% - 0.75%) in the surgical population. The mean number of degenerated lumbar discs was 2.1 ± 1.1. Compared to the 1,241 patients with isolated LDH, no investigated factors were significantly associated with tandem herniations.

Conclusion

From a genetic, pathophysiological, and epidemiological position, disc herniation is not commonly a consequence of disc degeneration. Rather, degeneration and herniation seem to exist as two separate and distinctly different processes. Based on the literature, it is tenable that tandem disc herniation does not deviate from the normal pathophysiology, but rather occurs in the rare case that two individual herniated discs coincide.

## Introduction

Lumbar disc herniation (LDH) and cervical disc herniation (CDH) represent an increasing public health problem, and lead to a considerable loss in quality-adjusted life years. Although disc herniation is well-known and documented, the pathophysiology is less well understood [1]. From a clinical point of view, a correlation between disc herniation and degeneration seems plausible [2]. While disc degeneration is a physiological, age-related process modified by several lifestyle and genetic factors, disc herniation usually occurs in a relatively younger population, and any related genetic influences are poorly understood [3-6].

Disc degeneration, as judged by magnetic resonance imaging (MRI), is common [4,7]. However, only a subset of degenerated discs herniate. Still less of these herniations do not spontaneously resorb and are symptomatic for long enough to require surgical intervention [8]. So-called tandem disc herniations, which require intervention in the cervical and lumbar spine, are rare in daily clinical practice. While there are some studies that screen patients for tandem spinal stenosis or disc degeneration, the literature contains no data on symptomatic tandem disc herniation [2-3]. It stands to reason that patients with simultaneous symptomatic disc herniation in two regions of the spine may show a certain genetic predisposition for disc herniation, or exhibit specific demographics.

We report a case series of 16 patients, which is, to the authors’ best knowledge, the first to describe patients with symptomatic tandem herniations of the cervical and lumbar spine that both required surgical intervention. In addition, we review the literature for other studies that looked at tandem degenerative changes, and describe the pathophysiology and genetics of intervertebral disc herniation and degeneration.

## Materials and methods

Overview

From a prospective institutional registry of all consecutive lumbar and cervical surgical procedures, all patients with simultaneous LDH and CDH were identified. All patients were operated on by two senior neurosurgeons at a specialized spine surgery center. Indications for cervical and lumbar discectomy were consistent: (1) presence of intractable, radiating arm or leg pain, (2) persistence for more than eight weeks without signs of improvement and (3) without improvement by nonsurgical treatment or analgesia, or (4) presence of myelopathy or (5) cauda syndrome. All patients underwent stand alone anterior cervical discectomy and fusion (ACDF) for CDH and tubular microdiscectomy (tMD) for lumbar disc herniation (LDH) as described previously [8]. In a few cases when patients presented with both symptomatic LDH and CDH, the surgeon gave way to operate the cervical herniation to prevent progressive clinical myelopathy. In addition, we assessed lumbar MRI imaging of patients with tandem disc herniations to determine the number of overtly degenerated lumbar intervertebral discs. Indicators of intervertebral disc degeneration were a decrease in signal intensity (“black disc”) with or without narrowing of the disc space. To identify factors associated with tandem disc herniations, characteristics were compared to a cohort of 1,241 patients with isolated LDH. These characteristics included patient gender, smoking status, age, body mass index (BMI), American Society of Anesthesiologists (ASA) grading, and location of the disc herniation. Thoracic disc herniation was also screened for. All included patients provided written informed consent. This registry was approved by the local institutional review board (Medical Research Ethics Committees United, Registration Number: W16.065), and this study was conducted according to the Declaration of Helsinki.

Statistics

Continuous variables are given as mean ± standard deviation, and categorical as numbers (percentages). Intergroup differences were assessed by continuity-corrected Mann-Whitney U and Pearson chi-square tests, depending on data type. The prevalence of tandem disc herniations was estimated with a 95% confidence interval (CI), under the assumption of a normal distribution. Analyses were carried out using R Version 3.4.3 (The R Foundation for Statistical Computing, Vienna, Austria). A *p* ≤ 0.05 was considered statistically significant.

Review of the literature

We searched MEDLINE and Embase for tandem herniation and degeneration with a range of keywords including “intervertebral disc”, “herniation”, “degeneration”, “multilevel”, “cervical AND lumbar”, “genetics”, and screened reference lists of relevant articles.

## Results

Patients

A consecutive series of 3,156 patients were operated between January 2011 and January 2018 for LDH or CDH, and prospectively recorded in our institutional registry. No cases of thoracic disc herniation were identified. Out of these, 16 patients presented with symptomatic tandem LDH and CDH. All of these patients required and underwent surgery for both disc herniations. We estimate the incidence of symptomatic tandem disc herniations in a population of patients with symptomatic disc herniation to be at 0.51% (95% (CI): 0.26% - 0.75%). No patient had undergone previous spine surgery. Table 1 reports individual patient characteristics.

**Table 1:** Summary of individual demographic and surgical patient characteristics BMI: body mass index; ASA: American Society of Anesthesiologists; mos: months; M: male; F: female.

Patient No.	First operated level	Second operated level	Gender	Active Smoker	Age	BMI	ASA Class	Number of degenerated lumbar discs	Time between surgical procedures [mos.]
1	C5-C6	L5-S1	F	no	42	32	I	1	8
2	C5-C6	L5-S1	F	no	36	28.4	I	1	49
3	C5-C6	L4-L5	F	no	43	22.9	I	2	46
4	C5-C6	L3-L4	F	no	50	23	I	2	55
5	C7-T1	L3-L4	M	no	46	26	I	2	10
6	C5-C6	L5-S1	M	yes	69	22.5	I	5	58
7	C6-C7	L5-S1	F	no	43	22.8	I	3	10
8	C5-C6	L4-L5	F	no	48	30.5	II	2	19
9	C4-C5	L5-S1	M	yes	56	28.7	II	2	5
10	C5-C6	L5-S1	F	no	38	21.3	I	1	4
11	C5-C6	L5-S1	F	no	48	24.5	I	3	8
12	L4-L5	C5-C6	M	no	39	28.3	I	3	15
13	L4-L5	C6-C7	F	no	48	30	I	1	18
14	L4-L5	C5-C6	F	yes	44	27	I	1	21
15	L4-L5	C6-C7	M	yes	39	23	I	1	3
16	L4-L5	C6-C7	M	no	48	23	I	3	13

Surgical data

Overall, disc herniations were most common at C5-6 with 10 patients (63%), and at L4-L5 and L5-S1, both with seven patients (44%). We observed more left-sided (75%) CDH and LDH. In a few cases when patients presented with both symptomatic LDH and CDH, the surgeon gave way to operate the cervical herniation to prevent progressive clinical myelopathy. This may explain why most patients received cervical spine surgery first. Patients with tandem disc herniations featured an average of 2.1 ± 1.1 degenerated lumbar intervertebral discs, with only 6 patients (38%) showing degeneration of just one isolated lumbar disc. Surgical time was 65.5 ± 26.2 for ACDF and 38.8 ± 13.8 for tMD. After surgery, patients stayed for a mean of 28.0 ± 3.5 hours after ACDF and 25.8 ± 2.6 hours after tMD. The mean time between the two surgical procedures was 21.4 ± 18.5 months.

Complications and reoperations

In 32 surgical procedures in this case series, a single case of recurrent laryngeal nerve injury after ACDF was the sole observed complication (3.1%). The complication led to transient hoarseness that improved throughout the first postoperative year. With a clinical follow-up duration of 4.4 ± 2.1 years, no patient has had to undergo re-discectomy. Patient number three was the sole patient requiring reoperation, and underwent laminectomy for stenosis at L3-L4.

Factors associated with tandem herniations

Characteristics of patients with tandem herniations were contrasted with 1,241 patients who had isolated LDH (Table 2). We compared gender, smoking status, age, BMI, American Society of Anesthesiologists (ASA) class, as well as disc herniation locations. No demographic and clinical factors exhibited statistically significant association with tandem CDH and LDH (all *p* > 0.05).

**Table 2:** Characteristics of patients with tandem cervical and lumbar, and with isolated lumbar disc herniations LDH: lumbar disc herniation; BMI: body mass index; ASA: American Society of Anesthesiologists

	Type of Disc Herniation		
Characteristic	Tandem (n = 16)	Isolated (n = 1241)	P
Male Gender	6 (38%)	661 (53%)	0.317
Active Smoker	4 (25%)	363 (29%)	0.864
Age [yrs.]	46.1 ± 7.7	44.8 ± 11.8	0.616
BMI [kg/m^2^]	25.3 ± 3.4	25.4 ± 3.5	0.329
ASA Class I	14 (88%)	881 (71%)	0.242
Location of Disc Herniation			
Right-sided	4 (25%)	566 (45%)	0.170
Median	1 (6%)	58 (5%)	0.999
Far-Lateral	1 (6%)	53 (4%)	0.999

## Discussion

In a registry of patients operated for disc herniation, only 0.51% presented with tandem cervical and lumbar disc herniation both of which required surgical intervention. In our small sample of 16 patients, no demographic or clinical factors exhibited statistically significant association with tandem disc herniations. To the authors’ best knowledge, this case series is the first to report on the phenomenon of symptomatic tandem disc herniations that necessitate surgery. 

Tandem disc herniation

Tandem herniations appear to be a rare clinical condition considering the low prevalence in our database of patients that were treated surgically for disc herniation. This is reflected in the literature, where we could not find a single study or case report. The age-related prevalence of asymptomatic disc herniations in the general population is estimated at 29% to 43% [9]. This demonstrates that disc herniation is common, but rarely the cause for intervention. Therefore, the chance of developing a second asymptomatic herniation is much higher than developing a second symptomatic herniation.

Considering these factors, we disagree with authors who claim that disc degeneration usually proceeds disc herniation, a plausible concept that lacks scientific evidence [1-2,10]. In fact, this has been debated before by Lama et al., who inferred that a high nucleus pressure is mandatory for disc herniation to occur, and that these high nucleus pressures can only be attained by non-degenerated discs [1]. Schroeder et al. find that discectomy leads to a higher rate of subsequent disc degeneration [10]. However, this finding may simply coincide with the demonstrated natural history of disc herniation, as extrusion of large amounts of healthy nucleus pulposus tissue through a ruptured annulus lead to loss of disc height and degenerative changes in the nucleus of the index level, as well as to a change in spinal biomechanics that may lead to adjacent segment degeneration [11-12]. This agrees with the evidence on traumatic disc herniation, whereas back injury does not seem to play a role in the development of disc degeneration, as Hancock et al. showed in a twin study [13]. Disc degeneration is estimated to be genetically determined between 61% and 73% [4-5,14-16]. The data on genetic susceptibility for disc herniation, on the other hand, is very scarce. Only two studies describing a correlation of specific polymorphisms of collagen type XI and thrombospondin-2 with annular rupture exist [6,17]. If the genetic predisposition for herniation were similar to that of degeneration, one would also expect a higher prevalence of tandem herniations. In general, patients with symptomatic disc herniation present in their second to fourth decade of life, whereas problems pertaining disc degeneration increase linearly with age [2,9,18-19]. These findings infer that disc herniation is not usually the result of disc degeneration.

The pathophysiology of intervertebral disc herniation is not well understood, and no clear disease model is available. However, from the literature, there are some predisposing factors which can be gleaned. *In vitro*, only mildly degenerated discs herniated when subjected to 100.000 axial load cycles compared to more degenerated discs [20]. Furthermore, disc herniation occurs earlier when applying compression combined with axial rotation, then, the posterolateral inner annular zones of the disc show high-stress peaks and centripetal pressure gradients [21]. This might explain why static axial overloading primes lumbar caprine discs for posterior herniation [22-23]. *In vivo*, it is generally the junction between the annulus and endplate that fails, in agreement with the failure modes found *in vitro* [24]. High intradiscal pressures, which can be expected after prolonged unloading during spaceflight, might explain the increased risk for herniation that astronauts face when returning from long-term orbit [25-26]. A longitudinal study found that the disc height is related to the size of the herniation [11]. Over time, the size of the initial herniation is predictive of the amount of disc height loss over time, which indicates that nucleus content which herniated from the disc was functional tissue, as opposed to degenerated tissue. These are further indications that strengthen the notion that disc degeneration and true disc herniation are different entities.

We propose that disc herniation is the result of a protrusion of relatively healthy nucleus tissue through a degenerated or mechanically compromised annulus, more specifically the annulus-endplate interface. There is little evidence on what might degenerate the annulus, but the aforementioned *in vitro* work suggests that overloading might play a role [22,27]. This is reflected in epidemiological data which shows relatively little disc degeneration occurring due to occupational or habitual overloading but that overloading definitely increases the chances of developing disc herniation [16]. Infection, especially with Propionibacterium acnes, has been named a possible cause of annular degeneration and herniation, and might weaken the annulus, particularly in immunocompromised patients [28]. However, this hypothesis does not explain why disc herniation most often occurs at a single level, and more research in larger cohorts is needed.

Tandem disc degeneration

While there is very limited evidence on tandem herniated discs in the peer-reviewed literature, a few studies examine simultaneous lumbar and cervical disc degeneration. Multiple radiological reports discuss multi-segment disc degeneration, which appears to be age-related [2-3,18,29-30]. While these identifiable degenerative changes seem to occur concurrently in the lumbar and cervical spine in 24% to 79%, virtually all are asymptomatic [3,9,29-30]. In general, the prevalence of lumbar and cervical disc degeneration in the normal population is estimated at up to 90%, and increases linearly with age [7,9,29]. Of these, only a fraction demonstrate compression of neural structures through bulging [7,29]. Thoracic disc degeneration as judged on MRI occurs less frequently, but is strongly associated with cervical disc degeneration [30]. This finding underlines the notion that disc degeneration is generally a multi-segment disease, while disc herniation usually presents at a single level. The genetic profile of disc degeneration further corroborates this.

Up to 73% of the variance in disc degeneration is explained by genetics [4-5,14-16]. Specifically, Annunen et al. identified a disease-causing sequence variation in collagen type IX alpha 2 chain (COL9A2), a gene which codes for a polypeptide chain of collagen IX [14]. Seki et al. found that a single nucleotide polymorphism (SNP) in the cartilage intermediate layer protein (CILP) gene plays a causative role in the development of disc degeneration [15]. An analysis of genome-wide association and meta-analysis of 4,683 individuals by Williams et al. found that methylation of the promotor for the Parkinson protein 2 (PARK2) gene is strongly associated with lumbar disc degeneration [5]. In fact, Battié et al. argue that less than 5% of the variance in back pain-causing disc degeneration is explained by environmental factors. Instead, changes in expression of structural and degradative proteins largely accounted for the variance [4,16].

Thus, when comparing intervertebral disc degeneration and lumbar disc herniation from a genetic, pathophysiological, and epidemiological point of view, they represent two different pathophysiological entities. However, it is a clinically viable idea that both diseases are related, and may thus occur simultaneously [2]. Finally, it is of clinical importance to differentiate neural compression through true disc herniation, with extrusion of relatively healthy nucleus pulposus tissue through a degenerated annulus, and through disc bulging by a degenerated disc with height loss.

Propensity or coincidence?

Based on our data, we estimate the prevalence of symptomatic tandem disc herniations at 0.51% of the surgical population, acknowledging that the incidence of tandem disc herniations in the general population will be even much lower. This extremely low incidence, combined with the literature on pathophysiology and genetics, makes it unlikely that tandem disc herniation has a deviating underlying pathophysiology. Rather, it is conceivable that tandem disc herniation simply occurs in the rare event that two individual symptomatic herniated discs coincide. Therefore, it is unlikely that patients with symptomatic tandem disc herniation will require special treatment. Studies with larger sample sizes and regular follow-up would be needed to further examine the clinical specifics of tandem herniation. Due to the low incidence of symptomatic tandem disc herniations, such efforts can only be successful by implementing large, multicenter registries.

While, based on the literature, it seems reasonable to assume that cervical and lumbar disc degeneration are heavily correlated, there is limited evidence on symptomatic disc herniation occurring concurrently in the cervical and lumbar spine. Furthermore, disc degeneration and disc herniation likely are two distinctly different processes. This is also supported by the fact that, in some patients, there were multiple years of difference between the cervical and lumbar surgeries. The differences between the two in terms of pathophysiology, associated genetic propensities, and epidemiology support this notion. It is important to differentiate neural compression through true disc herniation, with extrusion of relatively healthy nucleus pulposus tissue through a degenerated annulus, and through disc bulging by a degenerated disc with height loss.

Limitations

Although we analyzed a very large and complete database, only 16 patients with tandem herniations could be included. It is possible that some patients with tandem disc herniations may have been lost to follow up because they underwent surgical treatment elsewhere. This provides for a difficulty in performing a meaningful statistical analysis with adequate power, a problem which we aimed to tackle by using strict statistical methods. This was a single-center study. The incidence and characteristics of tandem disc herniation in other demographics may thus differ from those observed at our center. Furthermore, some patients had multiple years of latency between the first and the second symptomatic herniation. “True”, simultaneously symptomatic and treatment-requiring tandem herniation is even more infrequent than the “dual” disc herniations that the majority of our patients experienced, with a substantial latency between symptoms. Lastly, we can make no claims as to the prevalence of asymptomatic tandem disc herniation.

## Conclusions

Tandem disc herniation is an exceedingly rare phenomenon, especially when symptomatic, and occurs in around 0.26% to 0.75% of patients with disc herniation. Based on the literature, it is tenable that tandem disc herniation does not deviate from the regular pathophysiology, but rather occurs in the rare case that two individual herniated discs coincide. Furthermore, from a genetic, pathophysiological, and epidemiological position, disc herniation represents a different pathophysiological entity than intervertebral disc degeneration.
